# An Expanding Role of 2′,3′-Cyclic Nucleotide Monophosphates in Bacteria

**DOI:** 10.1021/acscentsci.2c01184

**Published:** 2022-11-15

**Authors:** Christopher Vennard, Herman O. Sintim

**Affiliations:** † Department of Chemistry, Purdue University, 560 Oval Drive, West Lafayette, Indiana 47907, United States; ‡ Institute for Drug Discovery, Purdue University, 720 Clinic Drive, West Lafayette, Indiana 47907, United States; § Purdue Institute of Inflammation, Immunology and Infectious Disease, West Lafayette, Indiana 47907, United States

**Figure 1:**
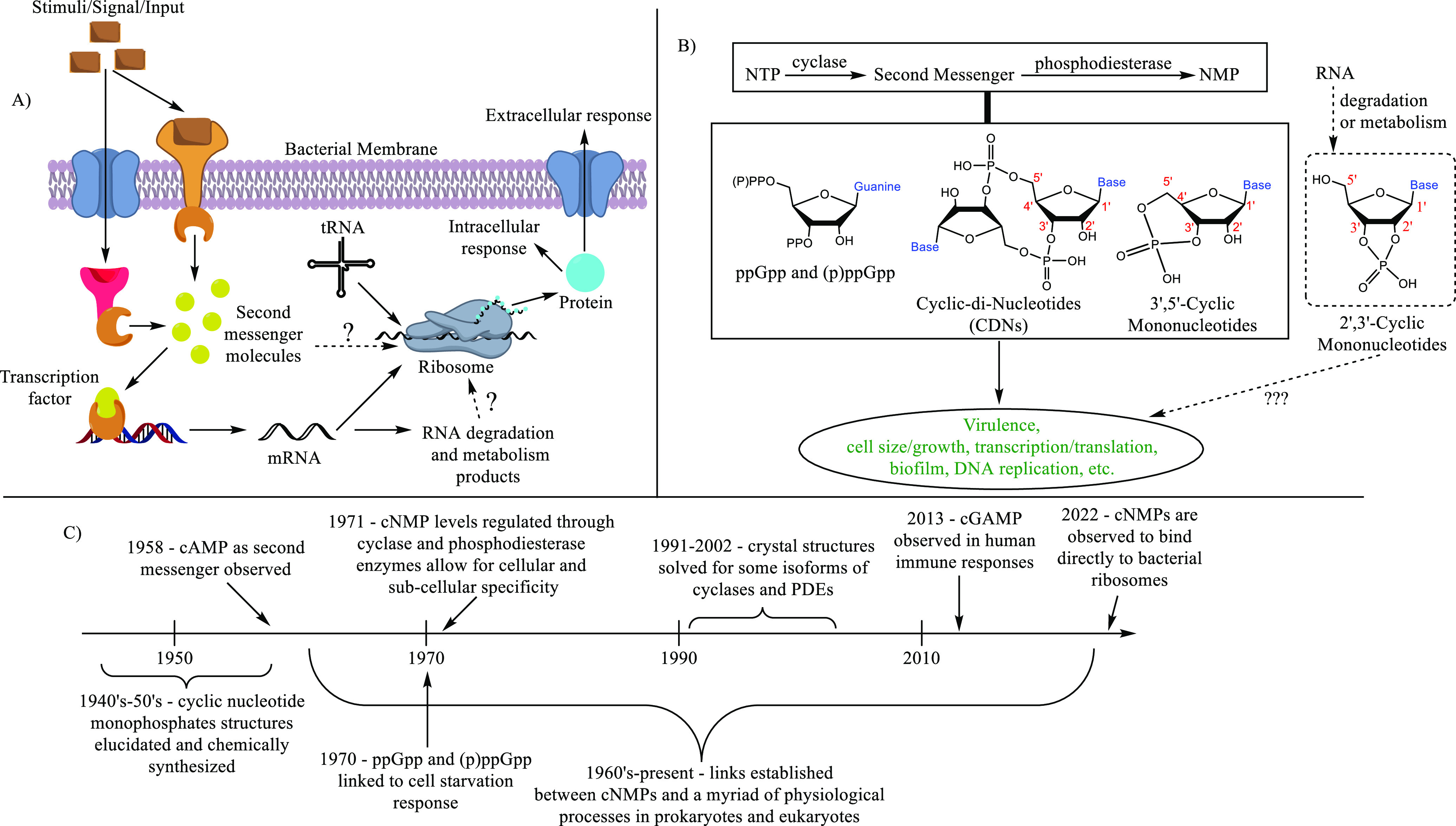
(A) A cartoon depicting a generic cell signaling pathway involving second messenger molecules. (B) Structures of biologically relevant nucleotide signaling molecules and their general physiological effects. (C) A timeline of important discoveries in cNMP signaling.

All organisms share the ability to perceive and subsequently react to their environment, including nutrient availability, via extracellular and/or intracellular receptors (Figure 1A) that bind to the first signals and relay the information to downstream effector systems. Additionally, organisms regulate key biological processes depending on the growth phase and/or cell density. A pioneering report by Sutherland and Rall almost 65 years ago revealed that extracellular hormones regulate mammalian cellular physiology via the production of 3′,5′-cyclic adenosine monophosphate (3′,5′-cAMP) second messenger.^1^ 3′,5′-cAMP and 3′,5′-cGMP form the paradigmatic nucleotide second messengers, which control many processes in both eukaryotes and prokaryotes.^2^ Other nucleotide signals, such as c-di-GMP/c-di-AMP/cGAMP and (p)ppGpp/(p)ppApp (Figure 1B for some structures), have also been identified as signaling molecules, and they have been shown to play key roles in both prokaryotes and eukaryotes.^3^ These nucleotide signals are typically produced from nucleotide triphosphates via the enzymatic action of synthases or cyclases, which become activated upon some environmental cue. 2′,3′-cyclic nucleotide monophosphates (2′,3′-cNMPs), positional isomers of the paradigmatic 3′,5′-cNMPs, have now been identified in all kingdoms.^4^ 2′,3′-cNMPs are not new *per se* as 2′,3′-cAMP and other 2′,3′-cNMPs were identified in *Escherichia coli* as far back as 1961 (around the same time that Sutherland described the biological effects of the analogous 3′,5′-cAMP regioisomer^1^).^5^ However, researchers had largely ignored 2′,3′-cNMP nucleotide signals, which now appear to be ubiquitous in all kingdoms, and it is only in the past few years that insights into how the 2′,3′-cNMPs regulate physiological processes have begun to emerge.

Unlike the well-established 3′,5′-cNMPs, which are synthesized from nucleotide triphosphates upon environmental stimulation, 2′,3′-cNMPs are thought to arise from RNase-mediated RNA metabolism.^4^ 2′,3′-cNMPs are further metabolized into 3′-NMP or 2′-NMP by 2′,3′-cNMP phosphodiesterases. The 3′,5′-cNMP system is well understood, and many blockbuster drugs that regulate the intracellular concentration of 3′,5′-cNMP have been developed. In contrast, there is a paucity of reports that describe effector systems that respond to changing concentrations of 2′,3′-cNMP. Thus, medicinal chemistry efforts to perturb 2′,3′-cNMP signaling have not started, given the dearth of knowledge about how 2′,3′-cNMP is regulated and its effector systems. In this issue of *ACS Central Science*, Weinert and co-workers demonstrate that 2′,3′-cNMPs, but not other nucleotides, bind to bacterial ribosomes and inhibit translation *in vitro*. The authors also showed that fluctuating levels of 2′,3′-cNMP can affect *E. coli* growth rates.^6^ This study expands our understanding of how the oft-forgotten 2′,3′-cNMP nucleotide signals affect physiological processes in bacteria and uncovers a potential strategy to develop novel antibiotics that affect translation.

Our understanding of 3′,5′-cNMP-mediated processes is significantly more than what is known about the less studied 2′,3′-cNMP system. 3′,5′-cAMP and 3′,5′-cGMP second messengers play outsized roles in mammalian systems, whereby they modulate a plethora of processes.^7^ In mammals, cyclic-nucleotide-gated ion channels, protein kinases, and exchange factors directly activated by cAMP (EPAC) are some of the protein targets that bind to 3′,5′-cNMP. In bacteria, 3′,5′-cAMP binds to the transcription factor cyclic AMP receptor protein (CRP) to regulate several genes in Gram negative bacteria,^2,7^ whereas 3′,5′-cGMP regulates encystment in cyst-forming α-proteobacteria.^2^ The role of 2′,3′-cNMPs in bacteria is, however, not clear. In 2014, Yan and co-workers rediscovered 2′,3′-cNMP in bacteria and reported the presence of 2′,3′-cCMP and 2′,3′-cUMP in *Pseudomonas fluorescences* pfo-1.^8^ The Yan group investigated if exogenously added 2′,3′-cNMPs affected bacterial biofilm but did not see any meaningful impact on biofilm formation. It is likely that the exogenous 2′,3′-cNMP could not penetrate into the bacteria. The Weinert group set about to identify binders of 2′,3′-cNMPs in bacteria.^6^ Since there is a lack of information regarding motifs that bind 2′,3′-cNMPs, the authors used an unbiased strategy to identify binding partners to 2′,3′-cNMP: they generated 2′,3′-cNMP linked Sepharose resins via the coupling of various 2′,3′-cNMPs with epoxy-activated Sepharose beads and used this to identify proteins in *E. coli* and *Salmonella typhimurium* that bind to 2′,3′-cNMPs via pull-down and subsequent mass spectrometry analysis. The majority of ribosomal proteins were identified in the pull-downs, suggesting that 2′,3′-cNMPs might bind to ribosomes. To validate ribosomes as *bona fide* binders of these nucleotides, the authors used 2′,3′-cGMP bound resin to sequester purified *E. coli* 70S ribosomes and showed that the bound ribosomes could be eluted with 2′,3′-cGMP (three times more than ribosome eluted with buffer alone without 2′,3′-cGMP). The authors used 2′,3′-cGMP as it was the only cNMP that was found to bind all ribosomal proteins. Considering that the cNMPs could bind to ribosomal proteins, the authors evaluated their effects on translation. Using an *in vitro* protein synthesis platform, which utilizes NanoLuc mRNA, Weinert and co-workers showed that 2′,3′-NMPs, but not linear analog 2′- or 3′-GMP or c-di-GMP, could inhibit translation at millimolar concentrations. It might be premature to extrapolate that the *in vitro* ribosome inhibition would translate to *in vivo* conditions. But it appears that at least under *in vitro* conditions, 2′,3′-cNMPs could inhibit protein synthesis. The authors used genetic means to modulate the intracellular levels of 2′,3′-cNMPs and showed that increased levels of the nucleotides resulted in slightly faster growth during the exponential phase but decreased cell density at the stationary phase. One has to be careful and not over interpret that the differential effects of 2′,3′-cNMPs at exponential and stationary phases was mainly due to ribosome inhibition as it is possible that other factors could also be at play. Plausibly, increased 2′,3′-cNMP levels could also lead to increased 2′- or 3′-NMP levels, and it is difficult to untangle the effects of the cyclic from the linear metabolite without additional well-controlled experiments. Nonetheless, the demonstration that cNMPs affect translation in vitro could be an inflection point for the field of 2′,3′-cNMP. Sixty years after being described by Wade, the effector proteins that bind to 2′,3′-cNMP are being uncloaked.

The inhibition of translation by cNMP *in vitro* is quite intriguing, but many gaps in knowledge need to be filled before we can begin to tie in how 2′,3′-cNMPs regulate key processes in bacteria, *vide infra*. Other nucleotide signals are also known to affect translation, and it will be interesting to decipher where 2′,3′-cNMPs place in the hierarchical system, which includes key players such as (p)ppGpp. It is known that during stressful conditions, bacteria produce (p)ppGpp, which reduces ribosome biogenesis and directly inhibits translation initiation via GTPase IF2 binding.^9^ Do 2′,3′-cNMPs crosstalk with (p)ppGpp to inhibit translation? Another aspect of 2′,3′-cNMP signaling that needs further clarification is how specific environmental signals affect the synthesis or degradation of a specific 2′,3′-cNMP signal (A/U/C/G). While it is known that 2′,3′-cNMPs arise from RNase-mediated metabolism of RNA, identification of specific environmental or internal cues that lead to differential production of 2′,3′-cNMPs would provide a more granular understanding of this specific nucleotide signal. The pull-down assay done by Weinert and co-workers identified other potential binding proteins, and future studies that characterize these putative 2′,3′-cNMP binding proteins could shed some light on how 2′,3′-cNMPs affect over 500 transcripts in *E. coli*.

Efforts to identify new antibiotics are always welcomed, given that resistance to traditional antibiotics is on the rise. The ribosome is a validated antibiotics target, and current antibiotics either target the 30S subunit at the decoding site or the 50S subunit (at the peptidyl-transferase center).^10^ Considering that mutation to these sites is a known resistance mechanism, the identification of novel sites on the ribosome that could be targeted with small molecules could facilitate the development of novel classes of antibiotics. Structural data showing how 2′,3′-cNMPs bind to ribosomal proteins could inform the development of cell permeable small molecules that bind to a putative 2′,3′-cNMP ribosome binding site to inhibit translation.
